# Chemical Characteristics of Water-Soluble Ions in Particulate Matter in Three Metropolitan Areas in the North China Plain

**DOI:** 10.1371/journal.pone.0113831

**Published:** 2014-12-01

**Authors:** Xu Dao, Zhen Wang, Yibing Lv, Enjiang Teng, Linlin Zhang, Chao Wang

**Affiliations:** 1 China National Environmental Monitoring Centre, Beijing, China; 2 School of Resource and Environmental Sciences, Wuhan University, Wuhan, Hubei, China; 3 Department of Geography, University of California Santa Barbara, Santa Barbara, California, United States of America; The Ohio State University, United States of America

## Abstract

PM_2.5_ and PM_10_ samples were collected simultaneously in each season in Beijing, Tianjin and Shijiazhuang to identify the characteristics of water-soluble ion compositions in the North China Plain. The water-soluble ions displayed significant seasonal variation. The dominant ions were NO_3_^−^, SO_4_^2−^, NH_4_^+^ and Cl^−^, accounting for more than 90% and 86% to the mass of total water-soluble ions in PM_2.5_ and PM_10_, respectively. The anion/cation ratio indicated that the ion acidity of each city varied both between sites and seasonally. Over 50% of the ion species were enriched in small particles ≤1 µm in diameter. The [NO_3_^−^]/[SO_4_^2−^] ratio indicated that vehicles accounted for the majority of the particulate pollution in Beijing. Shijiazhuang, a city highly reliant on coal combustion, had a higher SO_4_^2−^ concentration.

## Introduction

Particulate matter (PM) in the atmosphere originates from either direct emission or physical and chemical transformation of gaseous pollutants [1]–[3]. Atmospheric aerosols have potential effects on human health, radiation balance, climate, and visibility [4], [5]. Air quality has become an increasing public health concern since a series of heavy pollutions of fine particulate matter (PM_2.5_, particles ≤2.5 µm in diameter) occurred in Beijing in 2011 winter. According to the WHO air quality guidelines and interim targets for particulate matter in developing countries, the 24–hour concentration of PM_10_ and PM_2.5_ should be controlled below 150 and 75 µg/m^3^, respectively [6]. US EPA set the safe PM_10_ and PM_2.5_ concentration at 50 and 35 µg/m^3^, respectively [7]. With the increase in public awareness of the degradation of visibility or hazy weather, China released the Chinese National Ambient Air Quality Standards (NAAQS) in 2012, taking 150 and 75 µg/m^3^ as the 24-hour PM_10_ and PM_2.5_ limits in urban area, respectively, and 70 and 35 µg/m^3^ as the annual PM_10_ and PM_2.5_ limits, respectively. Previous epidemiological research has shown that high levels of particulate pollution lead to increased morbidity and mortality, as well as respiratory symptoms [8]–[11]. Smaller particles are deposited more easily in the lungs [9], and are thus more harmful to health than larger particles [12]. The chemical composition of the particles is important for understanding atmospheric visibility [13], as well as the locations of deposition in the lung [14]. Water-soluble ions are a key area of atmospheric environmental research [15]. Water-soluble ions, such as NH_4_^+^, Na^+^, K^+^, Ca^2+^, Mg^2+^, SO_4_^2−^, NO^3−^, F^−^ and Cl^−^, are significant components of atmospheric particles [16], [17], which varies depending upon the particle source [18], [19].

North China is one of the regions in China with the highest frequency of haze events [20], [21]. According to the PM monitoring in Beijing during 2001 and 2003,the average 12-hour PM_10_ mass concentration reached as high as 263.2 µg/m^3^ and PM_2.5_ ranged from 107 to 181 µg/m^3^ [22]. Another research showed the average PM_10_ annual mass concentration were 124 µg/m^3^ in Beijing and 141 µg/m^3^ in Tianjin between 2009 to 2010 [23]. Both 24-hour and annual PM concentrations reported are far higher than the limits of NAAQS. Air pollution has increased as a function of population and the diversification of socio-economic activities. However, there have been few studies on PM_2.5_ and PM_10_ with respect to their chemical composition and seasonal variation [24]. The majority of research on this region has focused on the composition [25], seasonal variation [26] and source of the particulate matter [27]. Generally, carbonaceous aerosol is the most abundant component of PM_2.5_ mass, and the main ions were NH_4_^+^, SO_4_^2−^ and NO_3_^−^ [13], [26], [28]. Different dominant mechanisms for the formation of SO_4_^2−^, NO_3_^−^ and Cl^−^ are found in the summer and the spring [29]. Various aerosol speciations associated with PM pollution display a distinct seasonality [30]. Remote sources, which were primarily soil derived Ca^2+^, Mg^2+^ and Al, contribute a major part of the PM pollution in spring [13], [30]. Seasonal peaks of organic carbon were commonly found in winter due to the high usage of coal for heating in the region [13], [31]. Simulation showed that the PM_2.5_ concentration was sensitive to NH_3_ emission [32]. In general, water-soluble ion species, including Na^+^, NH_4_^+^, K^+^, Mg^2+^, Ca^2+^, Cl^−^, NO^3−^, and SO_2_^4−^, comprise 25%-50% of the aerosol mass predominantly in the form of sulfates, ammoniums and nitrates [33]. Water-soluble ions can scatter or absorb both incoming solar radiation and thermal radiation emitted from the Earth’s surface, thus directly change the radiation balance [34]. Previous studies mostly focused on a single size particle either in Beijing or in Tianjin [24], [35]. Only a few studies addressed PM concentrations across the major cities in North China Plain [36]. In addition, these studies seldom referred to the national atmospheric background status at the same period. This study, derived from a national atmospheric monitoring program in 2013, focuses on the water-soluble ion compositions and their variation in particulate matter in 3 metropolises in North China area. Also data from national atmospheric background sites are referred at the same sampling periods.

## Experiment

### 1. Sampling

To compare the levels of the particulates and water-soluble ions in three cities (Beijing, Tianjin, Shijiazhuang) and four national atmospheric background sites, PM_2.5_ and PM_10_ samples were collected simultaneously at the seven sites in each season during 2013. Sampling locations of 3 studied cities were at the downtown area, while the background sampling sites were at remote rural area. Meanwhile samples from a control site at Datong city, which is close to the study area, were also collected. The field studies were carried out in the monitoring stations controlled or supervised by the China National Environmental Monitoring Centre (CNEMC). CNEMC has authority to permit our access to each station. Our field studies only collected atmospheric samples, which did not involve any endangered or protected species. The sampling sites are summarized in Table 1. Beijing, Tianjin and Shijiazhuang are located at the northwestern edge of the Great North China Plain, surrounded by mountains in all directions, except the south. The mountains extend several hundred kilometers and the highest peak is greater than 3000 m in elevation. The considerable emission of air pollutants in the region produces a high concentration of atmospheric particles.

**Table 1:** Locations of the sampling sites.

City/Province	Site name	Location	Possible major PM contributors
Beijing (BJ)	National environmentalmonitoring station	116°27’36’’ E;39°55’12’’ N	PM_2.5_: secondary aerosols, coal and biomassburning [37]; PM_10_: Soil dust [27]
Tianjin (TJ)	Tianjin environmentalmonitoring station	117°12’0’’ E;39°7’48’’ N	Crust dust, vehicle exhaust, secondarysulfate and nitrate [38]
Shijiazhuang(SJZ)	Shijiazhuang environmentalmonitoring station	114°28’48’’ E;38°1’48’’ N	Coal and biomass combustion^a^
Jilin	Changbai Mountain(background site #1)	128°07’50’’ E;42°08’22’’ N	Forest release, sea-salt, A small amount ofbiomass combustion, Long distance transportation^b^
Shanxi	Pangquan river (background site #2)	111°28’14’’ E;37°50’43’’ N	Forest release, A small amount of biomasscombustion, Long distance transportation^b^
Hubei	Shennongjia Mountain(background site #3)	110°16’24’’ E;31°27’14’’ N	Forest release, A small amount of biomasscombustion, Long distance transportation^b^
Guangdong	Nanling Mountain(background site #4)	112°53’56’’ E;24°41’56’’ N	Forest release, sea-salt, A small amount ofbiomass combustion, Long distance transportation^b^
Shanxi	Datong EnvironmentalMonitoring station	113°17’ 4′’ E;40° 4′ 27’’ N	Coal and biomass combustion^c^

a Online news source: http://www.pm25.org.cn/GuoNei/731.html. Accessed 13 August 2014.

b Those sites are National Atmospheric Background Sites, located at remote rural areas. Possible PM contributors are similar.

c No scientific publication or news report was found. Contributors were estimated.

A low-flow rate sampler, TH-16A (Tianhong, Wuhan, China) was deployed at the three city sites, and operated at a flow rate of 16.8 mL/min. A middle-flow rate sampler, TH-150 (Tianhong, Wuhan, China) was deployed at the four background sites, with a flow rate of 100 mL/min. Sampling was carried out at ∼23-h intervals, and conducted in each season for 5–7 days. The four periods were Feb 27^th^ to Mar 10^th^ in spring, Jun 19^th^ to 30^th^ in summer, Sep 10^th^ to 29^th^ in autumn, and Dec 7^th^ to 29^th^ in winter. Windy and/or rainy days were avoided collecting samples. 32 samples for National background point and 20–28 samples for each city site were sampled in each season. To assess the ion enrichment in particulate matter of various sizes, daily 23-h integrated PM_1_, PM_2.5_, PM_10_, TSP (from10:00 am to 9:00 am the next day, local time) samples were collected with pure quartz fiber filters (MUNKTELL, Sweden) using a multi-stage sampler (Kalman, Hungary, Model KS-303.150.10/10/2.5/1+PAH7) in Beijing. Teflon filters (Pall International, Cat) were used to determine the concentrations by mass of the atmospheric particles, The filters were weighed with a microbalance before and after sampling under identical conditions in a temperature- and humidity-controlled room (20±1°C and 35±5% relative humidity).

### 2. Analysis

Each filter was transferred into a plastic jar with the particle collection side facing downwards, and then leached with 15-mL Milli-Q water (18.5 MΩ·cm^−1^) in an ultrasonic bath for 30 min at room temperature. After ultrasonication, the leachate was filtered through a PTFE syringe filter (Fisher brand, 0.22 µm) before being introduced into an ion chromatography system (ICS-2000, Thermo Fisher). An AS19 analytical column (4×250 mm, Thermo Fisher), KOH eluent generator cartridge (EGC II KOH, Thermo Fisher) and 50-µL sample loop were employed for the determinations of anions, which included F^−^, Cl^−^, NO_3_^−^, and SO_4_^2−^. The cations of interest, Na^+^, NH_4_^+^, K^+^, Ca^2+^, and Mg^2+^, were determined with the same IC system with a CS12A analytical column (4×250 mm, Thermo Fisher), a 50-µL sample loop and MSA (EGC II MSA, Thermo Fisher) as the eluent. The above-mentioned anions and cations were considered to be the total water-soluble ions in this study. The detection limit for major anions, including F^−^, Cl^−^, NO_3_^−^, SO_4_^2−^, Na^+^, NH_4_^+^, K^+^, Ca^2+^, and Mg^2+^, was <2 µg/L. The recovery fell in the range 81–107%, and the precision based on seven duplicate spike samples was 3%.

All obtained data were categorized according to where and which season they were collected. Further statistical analysis including mean/variance, Pearson correlation and linear fits were calculated in the statistical package of Matlab 2013b software. Then, ion balance was used to evaluate the acid-base balance of the aerosol particles. We converted the ion concentration by mass into micro-equivalents, as follows:
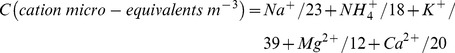
(1)




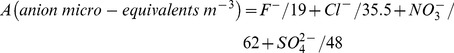
(2)


Micro-equivalents were also used for linear fits of secondary ions in Matlab 2013b software system.

## Results and Discussion

### 1. Seasonal variation of total water-soluble ions

Significant seasonal variation in the water-soluble compositions of PM_2.5_ and PM_10_ were identified in each city (Table 2). In spring, autumn and winter, the ions exhibited a similar concentration distribution, with the exception of a minor difference in SO_4_^2−^ in PM_2.5_ during spring. The order of the total ion concentrations in the three cities was Shijiazhuang> Tianjin> Beijing. The SO_4_^2−^ concentration in spring in the PM_2.5_ of Tianjin was slightly higher than that of Shijiazhuang. However, an inverse distribution was found in the summer. The ion concentrations of Shijiazhuang were lower than those of Beijing and Tianjin. During the sampling period, the average PM_2.5_ and PM_10_ concentrations by mass of the whole studied region were 167 and 273 µg/m^3^, respectively. The concentrations of water-soluble ions in both PM_2.5_ and PM_10_ displayed a significant variation with season, in the order spring> winter> summer> autumn. This might be explained by the high proportion of coal burning for heating in spring and winter, and the frequent dust storms in spring [39].

**Table 2:** Concentration of water-soluble ions (mean ± standard deviation in µg/m^3^) at sample sites in each season.

Sample Time	Ion	Beijing		Tianjin		Shijiazhuang	
		PM_2.5_	PM_10_	PM_2.5_	PM_10_	PM_2.5_	PM_10_
**Spring**	F^−^	0.504±0.23	0.742±0.418	0.369±0.223	0.445±0.217	1.263±0.628	2.555±0.462
n(BJ) = 28	Cl^−^	9.604±6.635	10.759±6.996	14.048±10.662	16.49±11.427	22.453±9.79	29.851±12.152
n(TJ) = 20	NO_3_^−^	40.382±32.66	42.828±34.801	50.892±37.408	59.778±43.775	63.515±45.764	75.82±51.112
n(SJZ) = 24	SO_4_^2−^	17.105±13.995	20.694±19.693	37.698±39.189	48.347±46.978	34.086±19.109	48.75±19.775
	Na^+^	1.949±0.418	2.322±0.402	1.595±1.237	2.2±1.198	3.903±3.254	6.079±2.466
	NH_4_^+^	19.052±14.792	22.303±19.296	26.136±16.008	28.757±19.878	39.024±19.302	40.622±19.092
	K^+^	2.066±1.411	2.543±1.991	2.884±1.802	3.383±2.107	5.943±2.412	6.904±2.778
	Mg^2+^	0.253±0.202	0.516±0.31	0.405±0.386	0.695±0.352	1.167±1.164	2.007±0.799
	Ca^2+^	1.653±0.553	5.693±3.213	3.362±4.086	6.894±4.727	12.995±12.349	23.655±6.488
**Summer**	F^−^	0.227±0.114	0.266±0.069	0.141±0.176	0.175±0.114	0.14±0.104	0.201±0.123
n(BJ) = 28	Cl^−^	3.431±1.332	4.919±1.659	3.468±1.891	5.159±3.551	2.717±0.889	2.871±0.722
n(TJ) = 20	NO_3_^−^	26.943±8.018	42.545±16.835	27.168±14.273	34.105±15.562	18.753±12.94	21.62±12.015
n(SJZ) = 24	SO_4_^2−^	32.521±10.684	43.787±14.133	38.185±15.247	39.567±18.175	33.591±23.703	34.598±21.062
	Na^+^	1.373±0.185	3.185±0.919	2.117±0.789	2.954±3.385	4.232±0.911	4.422±0.77
	NH_4_^+^	10.103±2.857	14.162±5.221	10.967±4.813	11.059±5.732	9.412±6.331	9.942±5.48
	K^+^	2.148±1.18	3.54±0.588	2.296±0.857	3.014±1.744	1.104±0.715	1.251±0.717
	Mg^2+^	0.173±0.053	0.663±0.156	0.315±0.505	0.527±0.203	0.233±0.047	0.331±0.071
	Ca^2+^	1.156±0.379	4.051±1.291	2.26±3.526	3.368±1.54	2.375±0.272	5.286±1.277
**Autumn**	F^−^	0.009±0.006	0.051±0.037	0.048±0.039	0.232±0.152	0.782±0.399	2.111±0.985
n(BJ) = 20	Cl^−^	2.046±1.009	2.901±1.664	2.212±1.353	3.589±1.687	12.766±8.523	18.985±9.613
n(TJ) = 20	NO_3_^−^	15.434±9.381	25.772±17.114	19.75±17.835	24.78±21.969	23.812±12.604	32.393±15.127
n(SJZ) = 24	SO_4_^2−^	13.799±8.014	19.077±12.206	17.429±13.822	17.81±13.456	22.985±16.775	37.175±23.107
	Na^+^	0.686±0.635	0.843±0.474	0.496±0.137	0.759±0.283	1.42±0.657	2.056±1.202
	NH_4_^+^	8.019±5.335	10.11±7.26	9.896±8.339	11.266±8.861	14.379±8.691	15.311±8.603
	K^+^	1.059±0.423	1.453±0.536	1.244±0.755	1.404±0.794	2.948±1.524	3.552±2.081
	Mg^2+^	0.081±0.038	0.569±0.411	0.072±0.025	0.491±0.245	0.403±0.181	0.854±0.478
	Ca^2+^	0.575±0.244	3.334±1.247	0.5±0.112	4.417±2.174	4.457±1.814	11.989±5.961
**Winter**	F^−^	0.112±0.147	0.332±0.18	0.151±0.073	0.682±0.336	0.35±0.017	1.791±0.997
n(BJ) = 20	Cl^−^	6.479±4.124	7.183±4.575	10.57±5.087	16.992±5.905	9.999±2.824	17.284±6.686
n(TJ) = 20	NO_3_^−^	11.731±8.35	13.131±9.138	18.959±20.804	32.535±31.024	16.333±11.523	39.424±19.737
n(SJZ) = 24	SO_4_^2−^	7.986±4.289	9.355±5.056	23.537±24.238	40.886±36.039	17.797±17.35	40.301±23.238
	Na^+^	1.108±0.519	1.343±0.46	1.127±0.281	1.75±0.144	1.793±0.518	3.498±1.72
	NH_4_^+^	7.43±4.234	7.469±5.002	15.714±14.452	22.715±18.584	12.814±7.953	27.037±9.963
	K^+^	1.46±0.649	1.527±0.719	2.059±1.003	2.729±0.967	2.064±0.449	4.316±2.136
	Mg^2+^	0.141±0.078	0.34±0.088	0.136±0.034	0.558±0.156	0.205±0.017	0.941±0.468
	Ca^2+^	1.346±0.332	4.438±1.207	1.111±0.404	5.613±0.682	3.029±0.965	10.981±3.432

### 2. Ionic species

The proportions of each water-soluble ion also presented a clear pattern. The concentrations of the ions were: NO_3_^−^> SO_4_^2−^> NH_4_^+^> Cl^−^> Ca^2+^> K^+^> Na^+^> F^−^> Mg^2+^ in spring and autumn; SO_4_^2−^> NO_3_^−^> NH_4_^+^> Cl^−^> Na^+^> Ca^2+^> K^+^> Mg^2+^> F^−^ in summer; and SO_4_^2−^> NO_3_^−^> NH_4_^+^> Cl^−^> K^+^> Ca^2+^> Na^+^> F^−^> Mg^2+^ in winter. In PM_2.5_, the concentrations of NO_3_^−^, SO_4_^2−^, NH_4_^+^ and Cl^−^ were significantly higher than those of the remaining five ions, comprising 90.3–92.3% of the total mass of water-soluble ions in each season. The total concentrations of these four dominant ions exceeded 70 µg/m^3^ in all samples. PM_10_ had similar ion species characteristics to PM_2.5_. Four dominant ions contributed 86.1%-88.6% of the total mass of water-soluble ions in each season, ranging from 37–195 µg/m^3^. The concentrations of Ca^2+^, K^+^ and Na^+^ were comparatively low, with average concentrations of 2.90, 2.27 and 1.82 µg/m^3^, respectively in PM_2.5_ and slightly higher average concentrations in PM_10_. Mg^2+^ and F^−^ combined contributed less than 0.5% of the total mass of ionic species. The concentration of F^−^ at the Shijiazhuang site ranged from 0.014–2.436 µg/m^3^ in PM_2.5_ and from 0.046–3.473 µg/m^3^ in PM_10_, an average concentration more than threefold greater than those at Beijing and Tianjin.

Differences in the dominant ions were found between the study area and the background sites. The NO_3_^−^ concentration was similar to or even greater than the SO_4_^2−^ in the study area, compared higher concentration of SO_4_^2−^ than NO_3_^−^ at the background sites. Cl^−^ and Ca^2+^ concentrations were one order of magnitude greater than those of K^+^ and Na^+^, while they were similar at the background sites. To give a broader picture, ion concentrations in PM_2.5_ of other important city in eastern Asian were shown in Table 3. The concentrations of NO_3_^−^ and SO_4_^2−^ were similar between Beijing and Shanghai, while NO_3_^−^ concentrations were higher in Beijing than in Guangzhou. SO_4_^2−^ concentrations were similar between the two cities. The three eastern Asia cities outside China had smaller equivalent ion concentrations.

**Table 3:** Comparison of annual ion concentrations in PM_2.5_ among major eastern Asian cities (µg/m^3^)**.**

Ions	Beijingn = 96	Tianjinn = 80	Shijiazhuangn = 96	Datongn = 104	Guangzhou,China [40]	Shanghai,China [41]	Singapore[42]	Yokohama,Japan [43]	Seoul,South Korea, [44]
F^−^	0.24	0.18	0.91	0.13	–	–	–	–	–
Cl^−^	5.88	7.38	13.84	1.58	1.8	2.34	0.6	0.21	0.17
NO_3_^−^	25.06	29.1	36.75	3.31	7.8	22.2	0.9	1.9	5.17
SO_4_^2−^	17.08	29.64	31.89	6.26	18.1	26.7	5.0	4.9	5.77
Na^+^	1.34	1.61	3.27	0.45	2.2	0.53	0.6	0.68	0.12
NH_4_^+^	11.95	15.78	22.54	2.82	5.1	4.54	1.62	1.10	3.70
K^+^	1.7	2.33	3.59	0.68	0.9	0.27	0.53	0.16	0.33
Mg^2+^	0.17	0.24	0.68	0.13	-	0.20	0.07	0.12	0.04
Ca^2+^	2.58	1.83	7.79	1.21	0.4	1.56	0.29	0.76	0.14

**Figure 1:**
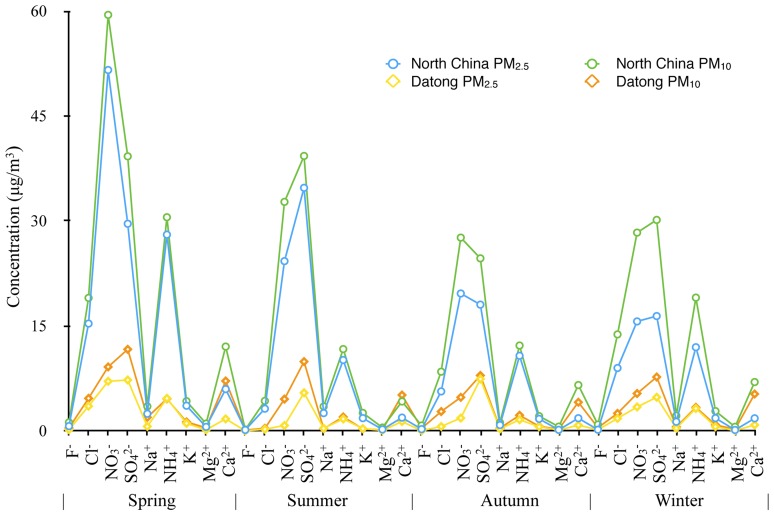
Seasonal variation of water-soluble ions in the study area and at the control point.

The Datong site was used as the control point. It is located in the upwind direction of the study area, and is isolated by the Taihang Mountains. Here too, the dominant ions were NO_3_^−^, SO_4_^2−^, NH_4_^+^, and Cl^−^. However, the concentrations were lower than those of the study area (Figure 1). The concentration of SO_4_^2−^ in spring was highest in Datong, 11.69 µg/m^3^ in PM_10_. The average concentration of SO_4_^2−^ in the study area reached 39.26 µg/m^3^, almost 3.4-fold greater than in Datong. The concentrations of the remaining ions in each sample were less than 10 µg/m^3^, significantly lower than in the study area.

Ion concentrations of study cities were divided by corresponding average ion concentration of 4 background sites. Namely, concentration multiples of study cities to background sites were used to calculate Pearson correlation coefficient for each site (Table 4). The water-soluble ion concentrations in PM_2.5_ were similar in the 3 case study cities (correlation coefficient>0.8334, *p*<0.05) and displayed relatively low similarity between 3 case study cities and Datong. This implied that the North China sites might have similar sources of PM_2.5_. There was a significant correlation between Datong and Shijiazhuang, and between Beijing and Tianjin in PM_10_ (correlation coefficient>0.8047, *p*<0.05). This implies that fugitive dust contributed a larger share of particles to Shijiazhuang. This is supported by the significantly higher Ca^2+^ concentration in Shijiazhuang, which is derived primarily from soil sources.

**Table 4:** Pearson correlation among study sites and control site by using seasonal average concentration multiples of study cities to background sites.

	Beijing	Tianjin	Shijiazhuang	Datong
Beijing		**0.8047**	**0.3387**	**0.2770**
Tianjin	0.9594		**0.5003**	**0.4566**
Shijiazhuang	0.8333	0.8334		**0.9120**
Datong	0.4783	0.4977	0.6758	

(The bold numbers were calculated based on PM_10_, and the regular numbers were based on PM_2.5_). (4 seasons, 9 ions, n = 36, p<0.05).

**Figure 2:**
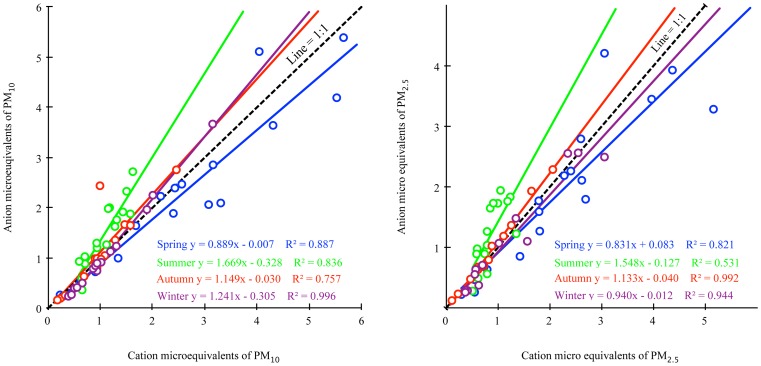
Total anions versus total cations. (All data were included and categorized by season, p<0.001 for all regressions.).

Correlations between the anion and cation equivalents were observed (Figure 2). Ion balance calculations stratified by season showed that summer and autumn samples were above the A/C (anion/cation) unity line in both PM_2.5_ and PM_10_, indicating that anion was excessive. In contrast, the spring anion was insufficient; i.e., below the A/C unity line. In winter, the ion balance relationships were inconsistent between PM_10_ samples and PM_2.5_ samples, indicating that the sources of PM_10_ and PM_2.5_ were different in winter. Higher regression slopes were found in PM_2.5_ samples. The reason might be the precursors of SO_4_^2−^ and NO_x_, which enriched primarily in the small particulate matter. A steeper slope was observed in summer primarily because SO_2_ and NO_x_ were more easily oxidized to H_2_SO_4_ and HNO_3_ and accumulated in the aerosols under the intensive illumination and high temperature [45]. A/C also varied by size and site location. Annual A/C ratios of PM_2.5_ in Beijing and in Shijiazhuang were smaller than 0.9, but larger than 1.1 in Tianjin. When coming to the PM_10_, Beijing and Tianjin were very close to 1.0. These results demonstrate that anions tend to be distributed in small particles.

### 3. Ion enrichment in particulate matter of various sizes

The size of the particulate matter affected its capability for ionic enrichment. The total mass concentration of the dominant ions contained in PM_2.5_ was about 60%-90% of the mass concentration of the dominant ions contained in PM_10_ (See table 2). That is, more ions were distributed in the fine particle (PM_2.5_) than that in the coarse particle (PM_2.5_–PM_10_). The enrichment of non-dominant ions displayed more complex relationships. In spring and summer, ∼50 −80% of F^−^ was found in PM_2.5_ at each site, while in autumn and winter the proportion dropped to 17–37%. The Na^+^ and K^+^ distributions were similar to those of the dominant ions, with an annual range of 59–96%, with the exception of Na^+^, which was present at a concentration of 43% in summer in Beijing. Greater complexity was displayed by the Ca^2+^ and Mg^2+^ distributions, both seasonally and spatially. <30% Ca^2+^ and <50% Mg^2+^ were found in PM_2.5_ in Beijing. That is, the source of Ca^2+^ and Mg^2+^ in Beijing was primarily large particles. High proportions of Ca^2+^ and Mg^2+^ in PM_2.5_ in spring and summer (∼49 −90%), and low proportions in autumn and winter (<25%) were found in Tianjin. The proportion of Ca^2+^ in PM_2.5_ in Shijiazhuang decreased from 55% in spring to 25% in winter. The proportion of Mg^2+^ in PM_2.5_ peaked in summer at ∼70%, and decreased to 23% in winter. Hence, the source of non-dominant ions might vary from site to site.

**Figure 3:**
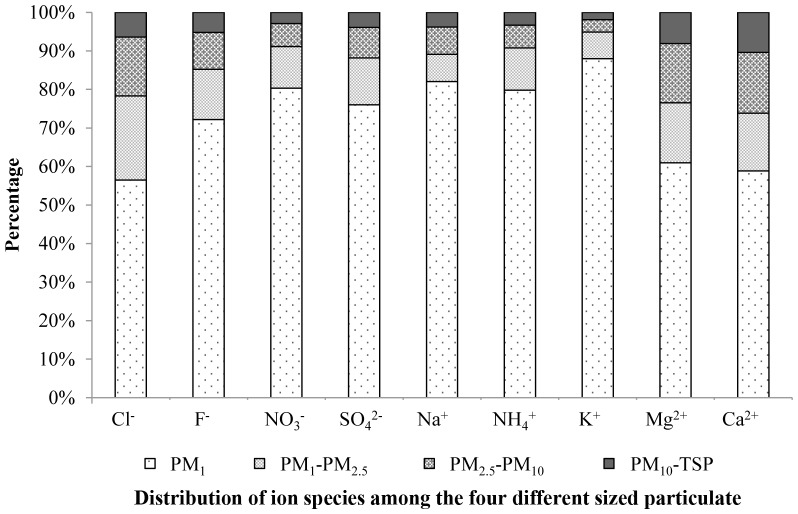
Ion enrichment in particulate matter of different sizes.

To investigate the ion enrichment of the different sizes of particulate matter, a further study was conducted in Beijing. We collected and analyzed PM_1_, PM_2.5_, PM_10_ and total suspended particulate matters (TSP). Figure 3 shows the concentrations of nine ions in particulate matter of different sizes. The smaller diameter particulate matter contained the highest concentration of soluble ions. For example, >56% of the water-soluble ions were distributed in PM_1_, compared to <10% in particles with a diameter>10 µm. Small particulate matter was more capable of carrying K^+^. While Cl^−^, Mg^2+^, and Ca^2+^ were present at higher concentrations in the larger sizes of particulate matter. Thus, controlling small particulate matter would substantially reduce the concentration of water-soluble ions in the atmosphere.

### 4. Secondary ions

Secondary ions play a key role in the formation of particulate matter [46]–[48]. The non-organic secondary ions NH_4_^+^, SO_4_^2−^ and NO_3_^−^ accumulate under stable weather conditions. Secondary ions also affect atmospheric visibility and human health [49]. The total mass of secondary ions contributed 42.4% and 31.5% of the PM_2.5_ and PM_10_, respectively. The major water-soluble ions were the secondary inorganic ions sulfate, nitrate and ammonium, which accounted for 77% of the total water-soluble ions in Beijing in 2008 [50]. The ratio of [NO_3_^−^]/[SO_4_^2−^] in aerosols can be used to analyze the contribution of mobile sources (such as vehicle exhaust) to stationary sources (such as boilers) [51]. Coal is the main source of SO_2_ emissions. NO_x_ originates from motor vehicles and coal burning [52], [53]. An increased NO^3−^ contribution to PM_1_ mass loading during polluted periods has been reported [54]. Table 5 shows the [NO_3_^−^]/[SO_4_^2−^] ratios of the different sites and seasons. The ratios during summer were smaller than those in the other three seasons because the high temperatures transform particulate nitrate into the gaseous state, thus reducing the [NO_3_^−^]/[SO_4_^2−^] ratio [55]. In winter, the [NO_3_^−^] <[SO_4_^2−^] in Tianjin and Shijiazhuang implies a dominance of sulfate pollution from heating and industrial coal burning. In contrast, in Beijing, [NO_3_^−^]> [SO_4_^2−^] and the concentration of NO_x_ continued to increase in Beijing in 2011 [56], which indicates that vehicles made a greater contribution to emissions than in Tianjin and Shijiazhuang.

**Table 5:** Seasonal [NO_3_^−^]/[SO_4_^2−^] ratios in three cities in the North China Plain in 2013.

NO_3_^−/^SO_4_^2−^	Beijing		Tianjin		Shijiazhuang	
	PM_2.5_	PM_10_	PM_2.5_	PM_10_	PM_2.5_	PM_10_
Spring	2.36	2.07	1.35	1.24	1.86	1.56
Summer	0.83	0.97	0.71	0.86	0.56	0.62
Autumn	1.12	1.35	1.13	1.39	1.04	0.87
Winter	1.47	1.40	0.81	0.80	0.92	0.98

The differences among the three cities reflect both the dramatic increase in vehicle numbers and the decreased usage of coal at the different stages of urban development (Table 6). Beijing had the largest contribution from vehicles, which was threefold greater than that of Shijiazhuang. Vehicles have already become one of the main contributors to particle emissions in metropolitans as the one of the main source of NO_3_^−^ [57]. For example, direct emission, road dust and secondary nitrates, which were closely related to vehicle use, contributed 27.9%-47.5% to total PM_2.5_ mass concentration in Beijing [37]. Larger transportation sector in Beijing and Tianjin consumed more petroleum, thus contributed more NO_3_^−^ emission. The industrial structure of the cities, which we did not investigate in depth, was the cause of the difference between coal and petroleum consumption. Coal was the main energy source, combustion of which results in release of both NO_3_^−^ and SO_4_^2−^. Shijiazhuang relied substantially on coal combustion, at a level almost threefold greater than that in Beijing. High coal combustion produces higher SO_2_ emissions, leading to higher SO_4_^2−^ concentrations. Therefore [NO_3_^−^]/[SO_4_^2−^] ratio here was close related to the energy use in the transport sector and the industrial sector.

**Table 6:** Vehicle amount and major energy consumption of 3 cities in 2012.

	Vehicle	Coal	Gasoline	Diesel
	10^6^vehicles	10^6^ tons	10^6^tons	10^6^ tons
Beijing [58]	5.20	22.70	4.16	2.16
Tianjin [59]	2.36	52.98	2.54	3.78
Shijiazhuang [60]	1.70*	60.07	0.91*	0.67

* Data were estimated.

Secondary ions have complex reactions. NH_4_^+^ readily reacts with SO_4_^2−^ to the stable form of ammonium salts [50], [61], [62]. The reaction of NH_4_^+^ and NO_3_^−^ is affected by the gas/aerosol distribution of the precursor gases (NH_3_ and HNO_3_) in terms of temperature and humidity [63]. [NH_4_^+^] was linearly correlated with the micro-equivalent sum of ([SO_4_^2−^]+[NO_3_^−^]) in the three cities. The linear fits of the data were:
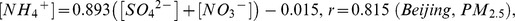





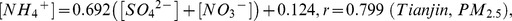





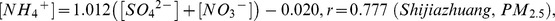





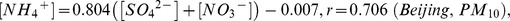





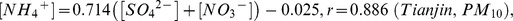





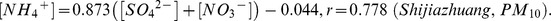



The PM_2.5_ regression slopes were <1 in Beijing and Tianjin, but slightly>1 in Shijiazhuang. The PM_10_ regression slopes of [NH_4_^+^] against ([SO_4_^2−^]+[NO_3_^−^]) at the three cities were <1, indicating that SO_4_^2−^ and NO_3_^−^ in large particles were incompletely neutralized. Larger slope coefficients and higher NH_4_^+^ concentrations were found in Shijiazhuang. One possible reason for this was the greater agricultural fertilizer usage (489.0 million tons in Shijiazhuang, compared to 136.7 million tons in Beijing and 244.5 million tons in Tianjin in 2012. Data from 2013 were not available at the time of our study), which was one of the primary sources of NH_4_^+^. Since evaporated ammonium was not taken into account, the extent of SO_4_^2−^ and NO_3_^−^ neutralization might have been underestimated.

### Conclusion

The concentration gradient of total water-soluble ions was in the order Shijiazhuang> Tianjin> Beijing. Spring had the highest concentrations. The total concentrations by mass for summer and winter were similar, and higher than those in autumn. The gradient of ion species across the North China region was NO_3_^−^, SO_4_^2−^> NH_4_^+^> Cl^−^> Ca^2+^, K^+^, Na^+^> Mg^2+^, F^−^. NO_3_^−^, SO_4_^2−^, NH_4_^+^, and Cl^−^ were the dominant ion species, contributing more than 90% and 86% of the concentration by mass of the total water-soluble ions in PM_2.5_ and PM_10_, respectively. The size of the particulate matter was an important determinant of the ion composition. Over 50% of the water-soluble ions were present in PM_1_, and 73.9%-94.9% in PM_2.5_. Secondary ions, including NO_3_^−^, SO_4_^2−^ and NH_4_^+^, comprised 43.4% and 31.5% of the total average concentrations by mass. The [NO_3_^−^]/[SO_4_^2−^] ratio indicated that the contribution of vehicle pollution increased with economic development. The anion/cation balance varied among sites and seasons, indicating that the source might be complex. The balance of secondary ions was likely influenced by the usage of agricultural fertilizer. The pollution patterns of the whole area were somewhat significantly correlated. Further studies of organic carbon and pollution source apportionment are needed.
